# Clinical validation and assessment of aortic hemodynamics using computational fluid dynamics simulations from computed tomography angiography

**DOI:** 10.1186/s12938-018-0485-5

**Published:** 2018-05-02

**Authors:** Yulei Zhu, Rui Chen, Yu-Hsiang Juan, He Li, Jingjing Wang, Zhuliang Yu, Hui Liu

**Affiliations:** 1 Department of Radiology, Guangdong General Hospital, Guangdong Academy of Medical Sciences, No. 106, Zhong Shan Er Lu, Guangzhou, 510080 Guangdong China; 2 School of Medicine, South China University of Technology, Guangzhou, 510006 Guangdong China; 3 Department of Medical Imaging and Intervention, Chang Gung Memorial Hospital, Linkou Chang Gung University, Taoyuan, Taiwan; 4 Department of Cardiac Surgery, Guangdong Cardiovascular Institute, Guangdong Provincial Key Laboratory of South China Structural Heart Disease, Guangdong General Hospital, Guangdong Academy of Medical Sciences, No. 106, Zhong Shan Er Lu, Guangzhou, 510080 Guangdong China; 5 College of Automation Science and Technology, South China University of Technology, 381 Wushan Road, Guangzhou, 510080 Guangdong China

**Keywords:** Multi-detector computed tomography angiography, Computational fluid dynamics, Aortic hemodynamics, Congenital heart disease

## Abstract

**Background:**

Hemodynamic information including peak systolic pressure (PSP) and peak systolic velocity (PSV) carry an important role in evaluation and diagnosis of congenital heart disease (CHD). Since MDCTA cannot evaluate hemodynamic information directly, the aim of this study is to provide a noninvasive method based on a computational fluid dynamics (CFD) model, derived from multi-detector computed tomography angiography (MDCTA) raw data, to analyze the aortic hemodynamics in infants with CHD, and validate these results against echocardiography and cardiac catheter measurements.

**Methods:**

This study included 25 patients (17 males, and 8 females; a median age of 2 years, range: 4 months–4 years) with CHD. All patients underwent both transthoracic echocardiography (TTE) and MDCTA within 2 weeks prior to cardiac catheterization. CFD models were created from MDCTA raw data. Boundary conditions were confirmed by lumped parameter model and transthoracic echocardiography (TTE). Peak systolic velocity derived from CFD models (PSV_CFD_) was compared to TTE measurements (PSV_TTE_), while the peak systolic pressure derived from CFD (PSP_CFD_) was compared to catheterization (PSP_CC_). Regions with low and high peak systolic wall shear stress (PSWSS) were also evaluated.

**Results:**

PSV_CFD_ and PSP_CFD_ showed good agreements between PSV_TTE_ (r = 0.968, p < 0.001; mean bias = − 7.68 cm/s) and PSP_CC_ (r = 0.918, p < 0.001; mean bias = 1.405 mmHg). Regions with low and high PSWSS) can also be visualized. Skewing of velocity or helical blood flow was also observed at aortic arch in patients.

**Conclusions:**

Our result demonstrated that CFD scheme based on MDCTA raw data is an accurate and convenient method in obtaining the velocity and pressure from aorta and displaying the distribution of PSWSS and flow pattern of aorta. The preliminary results from our study demonstrate the capability in combining clinical imaging data and novel CFD tools in infants with CHD and provide a noninvasive approach for diagnose of CHD such as coarctation of aorta in future.

## Background

Congenital heart disease (CHD) is a common malformation affecting approximately six per 1000 live births, occurring as an isolated trait or related to multiple congenital anomalies [1]. Despite anatomical evaluation has a great contribution to the diagnosis and treatments of CHD, the hemodynamic evaluation is also indispensable. Peak systolic pressure (PSP) and peak systolic velocity (PSV) were widely used in diagnosis or grading of CHD such as pulmonary arterial hypertension (PAH), aortic valve stenosis (AS), and coarctation of aorta (CoA) [2–4]. An accurate and noninvasive approach to evaluate such hemodynamic information may carry an important role to benefit the clinical diagnosis or grading of CHD.

Cardiac catheterization is the reference standard in obtaining patient-specific hemodynamic analysis, but is associated with patient discomfort and carries potential peri-procedural risks [5]. Transthoracic echocardiography (TTE) is the effective first-line technology for obtaining PSV, but is limited in the ability to provide accurate PSP information. Cardiac magnetic resonance (CMR) is another noninvasive method to evaluate hemodynamics in patients [6, 7], but it is expensive and more technically demanding. Currently, multi-detector computed tomography angiography (MDCTA) has been widely accepted as an accurate imaging modality to evaluate cardiovascular anatomy for its conveniences, noninvasive procedure and low cost; however, MDCTA cannot provide hemodynamic information directly. Recent studies showed that reliable hemodynamic results of carotid artery, coronary artery and other artery can be acquired using MDCTA raw data alone [8–12], but only few studies had focused on aortic hemodynamic schemes based MDCTA [13].

The aim of this study is to provide a noninvasive method based on a computational fluid dynamics (CFD) model, derived from multi-detector computed tomography angiography (MDCTA) raw data, to analyze the aortic hemodynamics in infants with CHD, and validate these results against echocardiography and cardiac catheter measurements.

## Methods

### Study population

We conducted a single center search of all patients with CHD from July 2015 to October 2016 in our department. Patients were included if they: (1) infants under 4 years old. (2) Had MDCTA and TTE studies, with flow measurement by TTE. (3) Cardiac catheterization within 2 weeks from MDCTA and TTE, with PSP measurement. (4) No malformation and lesion of aorta; Patients were excluded from this study if the studies had poor image quality, or if the above inclusion criteria were not met. There are twenty-five patients (17 males, 8 females) with known CHD were enrolled finally, with a median age of 2 years (range, 4 months–4 years).

### Transthoracic echocardiography, MDCTA and cardiac catheterization protocol

Before undergoing MDCTA, TTE (Philips iE 33 imaging system, Philips Healthcare, Andover, Massachusetts, USA) was performed.

A second-generation dual source CT scanner (Somatom Definition Flash, Siemens Health-care, Forchheim, Germany) was performed using electrocardiographic-gated step and shoot protocol. Short-term sedation was achieved with 0.1 mg/ml of oral chloral hydrate solution. The scans were performed in cranio-caudal direction from the thoracic inlet to the bottom of the heart. MDCTA parameters were as follows: 0.28 s gantry rotation time, 2 × 64 × 0.6 mm detector collimation, CARE kV (weight adapted setting for tube voltage and tube current). In the sequential mode, the acquisition window was set at 35–45% of the R–R interval. Safire (strength 3) is selected as the iterative reconstruction algorithm, the kernel is I26, the slice thickness is 0.75 mm, and increment is 0.5 mm. Iodinated contrast medium (Iopamidol, 300 mg I/ml, BRACCO, Italy) was injected intravenously at a volume of 1.5–2.0 ml/kg body weight, followed by 1.0 ml/kg body weight saline chaser with injection rate 1–2 ml/s. The acquisition delay was determined by the time of contrast medium entering both ventricles.

CC was performed by using Philips Allura Xper FD10 system (Philips Medical Systems, Best, the Netherlands) to get hemodynamics including PSP of the aortic isthmus (AI).

### Construction of aorta and mesh generation

**Fig. 1:**
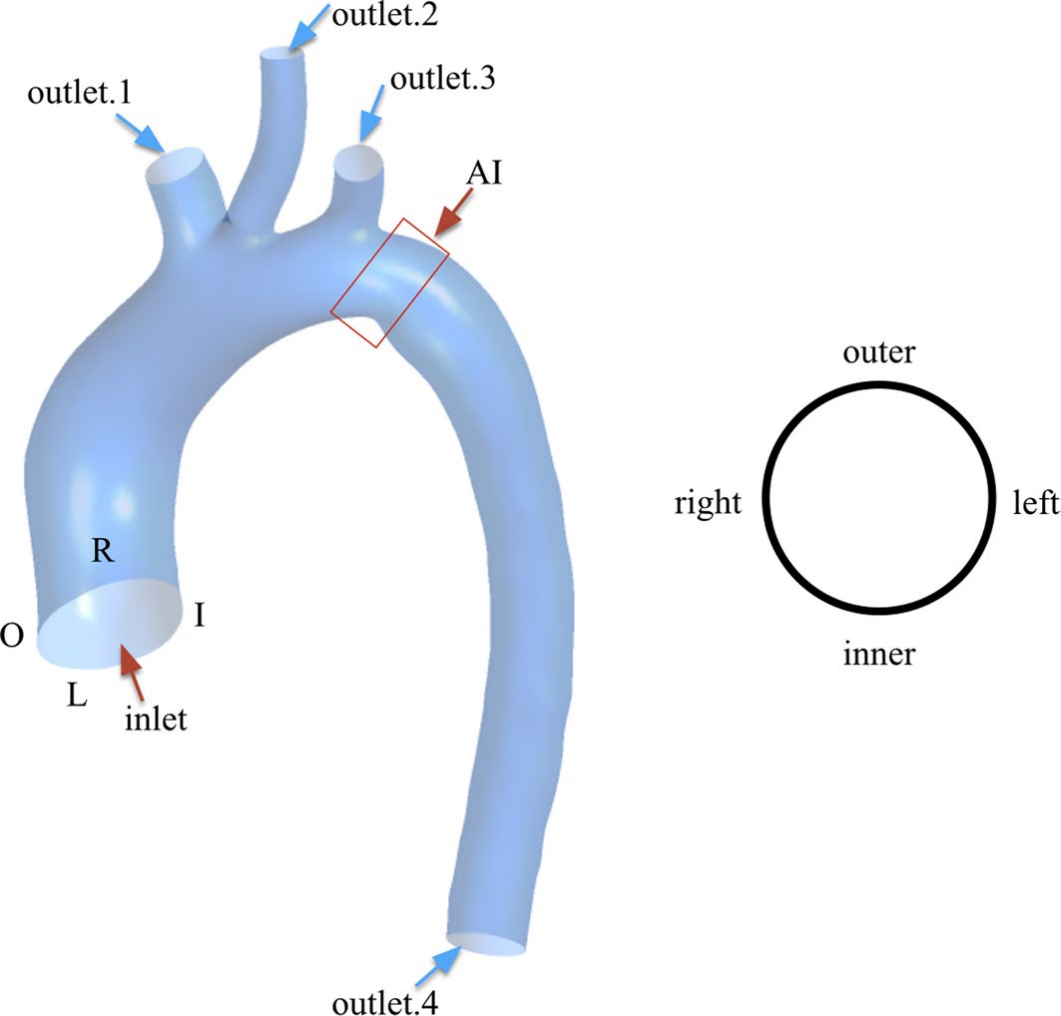
The patient-specific aortic geometry. An inlet boundary and four outlet boundaries were defined in left image. O, R, L and I represented the four partition of the aortic wall demonstrated in right image. The region identified by ‘AI’ was aortic isthmus

Computational representations of the aorta were created using Mimics 17 (Materialise, Leuven, Belgium) software that facilitates volume visualization and conversion of the MDCTA raw data into geometrically representative computer models, as demonstrated in Fig. 1. Models were discretized using an automatic mesh generation software (Ansys ICEM 14.5, ANSYS, Inc., Canonsburg, Pennsylvania, USA). The mesh generation followed a custom standard protocol with unstructured, formatted tetrahedral. In addition, prism mesh was added to the wall boundary (height = 1, ratio = 1.2, numbers = 3) in order to improve the accuracy of hemodynamic parameters adjacent to aortic wall. The total elements in each mesh ranging from 1,000,000 to 2,000,000.

### Boundary conditions

**Fig. 2:**
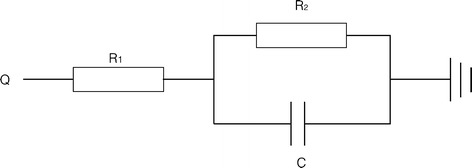
Scheme of LPM. *R*_1_ represented the characteristic resistance, *R*_2_ represented the peripheral impedance, and *C* represented the compliance of artery. *Q* was flow rate of artery, and pressure on outlet was calculated by solving the differential equation

Inflow and outflow boundaries were defined in Fig. 1, and the flow domain was defined as cavity of the reconstructed geometry. Velocity information in TTE data was mapped to the inlet of CFD models, while the blood pressure (BP) was used to prescribe the outlet boundaries. Lumped parameter model (LPM) was applied to confirm the outflow boundary conditions if BP of outlet was inaccessible. The parameter of LPM was shown in Table 1, and the schematic illustration of LPM was shown in Fig. 2. The unknown pressure (*P*) of outlet for CFD models was calculated from LPM, the equation of LPM was given as follow:

**Table 1:** Parameter of LPMs

Artery	R_1_ (mmHg s/ml)	R_2_ (mmHg s/ml)	C (ml/mmHg)
BA	0.100	2.480	0.466
LCCA	0.110	2.510	0.443
LSA	0.150	2.624	0.437
DAo	0.120	2.118	0.421

BA (brachiocephalic artery), LCCA (left common carotid artery), LSA (left subclavian artery), DAo (descending aorta). *R*_1_ was characteristic resistance, *R*_2_ was Peripheral impedance, and *C* was compliance of artery

1 $$P = (R_{1} + R_{2} )\,Q - R_{2} C\frac{dP}{dt} + R_{1} R_{2} \frac{dQ}{dt}$$

where Q was the volume flow rate through brachiocephalic artery (BA), left common carotid artery (LCCA), left subclavian artery (LSA), or descending aorta (DAo) respectively. R1 was the characteristic resistance of the artery, while R2 was Peripheral impedance of the artery, and C was the compliance of artery. The value of R_1_, R_2_, C were calculated according to previous work [13, 14]. The derivative items can be calculated using the backward Euler method:

2 $$P_{n + 1} = \frac{{(R_{1} + R_{2} + R_{1} \beta ) - R_{1} \beta Q_{n} + \beta P_{n} }}{1 + \beta }$$

3 $$\beta = R_{2} C/\Delta t$$

where *Δt* was time interval between P_n_ and P_n+1._

### Computational fluid dynamics simulation

Simulation was performed by using Ansys FLUENT 14.5 (Fluent Inc., Lebanon, New Hamshire, USA). In our calculation, there were some assumptions of physical properties of blood according to previous work: (1) Blood was assumed to be incompressible, viscous, Newtonian fluid. (2) The density (*ρ*) and viscosity (*μ*) of blood was assumed to be constant and equals to 1050 kg/m^3^ and 0.004 Pa s [14–17]. Calculation solved the Navier–Stokes equations as follow [18]:

4 $$\rho \left( {\frac{du}{dt} + u \bullet \nabla u} \right) = - \nabla P + \mu \nabla^{2} + F$$

5 $$- \nabla \bullet u = 0$$

where *u* was the blood velocity, *F* was body force equals to zero. Fluid structure interaction simulation was wildly used to illustrate the interaction between blood and vessels [19–21]. Since the hemodynamics of the blood is the main research topic in this current study, we have not included fluid structure interaction simulation into this study. Similar approaches had been applied in other related studies to acquire accurate hemodynamics from simulation without FSI [14, 22–24].

### Statistical analysis

The statistical analysis was performed by SPSS (SPSS 22, SPSS Inc., Chicago, USA). For continuous variables, data are expressed as the mean ± standard deviation (M ± SD). All tests were two-sided, and effects were considered significant at p < 0.05. Normality was tested using the Kolmogorov–Smirnov method, and variance homogeneity was then tested with the Levene’s test. Group differences were assessed by paired Student’s t test in normally distributed (Kolmogorov–Smirnov test) data. Otherwise, the paired Wilcoxon test was used.

To illustrate the accuracy of aorta reconstruction, morphometric parameters (diameters at three locations: aorta ascending, aorta descending, and stenosis) were analyzed by paired Student’s *t*-test. To demonstrate the accuracy of our simulation, the agreements between PSV_CFD_ and PSV_TTE_, PSP_CFD_ and PSP_CC_ were characterized with the Bland–Altman plot and linear fitting.

## Results

Table 2 summarizes the reconstructed and measured geometric parameters of the aorta. Mean reconstructed diameters of the ascending aorta (inlet), descending aorta (outlet.4) and AI were 19. 2 ± 6.0 mm, 10.9 ± 2.6 mm, and 12.1 ± 3.5 mm respectively. The paired *t* test found no difference between measured diameters and simulated diameters.

**Table 2:** Calculated and measured geometric parameters

Case	AscAo			AI			DAo		
	R	M	P	R	M	P	R	M	P
01	11.2	11.0	0.300	8.90	8.8	0.805	8.1	8.0	0.18
02	20.0	20.4		13.80	13.1		11.5	11.7	
03	25.8	25.0		19.34	19.0		16.1	15.0	
04	16.2	16.5		10.60	11.0		9.1	9.7	
05	28.9	29.5		17.40	17.5		14.5	14.5	
06	15.2	15.5		10.40	11.0		9.1	9.4	
07	21.2	21.5		13.00	13.2		11.8	11.4	
08	17.9	18.5		11.30	11.0		9.8	10.0	
09	17.7	18.2		12.80	13.0		12.6	12.0	
10	11.7	12.0		8.20	8.4		8.0	7.8	
11	19.4	20.5		10.80	11.0		10.0	10.0	
12	17.2	17.4		12.80	12.0		11.4	11.5	
13	17.1	17.0		14.24	14.5		12.3	12.0	
14	21.0	21.4		16.40	16.7		15.3	16.0	
15	18.0	18.4		9.70	10.7		8.9	8.4	
16	35.0	35.0		13.38	13.5		10.0	10.0	
17	11.1	11.0		7.80	6.9		7.0	7.7	
18	22.3	22.5		15.40	15.5		14.1	14.0	
19	26.7	26.5		13.40	12.8		10.7	10.3	
20	23.0	23.0		13.50	13.2		11.0	10.2	
21	26.1	26.4		17.20	17.4		16.0	15.4	
22	13.0	12.5		6.60	6.7		8.3	8.7	
23	11.6	11.0		5.30	5.5		8.4	8.0	
24	17.7	17.3		11.80	11.5		9.7	9.2	
25	16.2	15.6		10.10	9.7		8.6	8.2	

R, M, and P were represented the reconstructed aorta, measured aorta and p value

**Fig. 3:**
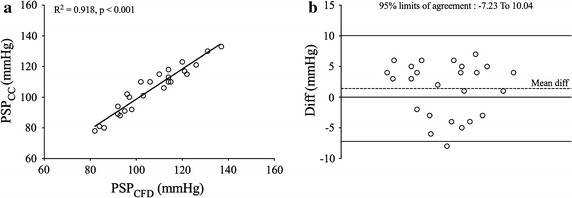
Validation of PSP derived from CFD. Demonstration of PSV and PSP validation. The image **a** was linear fitting of PSP, and image **b** was Bland–Altman plot of PSP. The reference line of Bland–Altman plots was mean difference ± 1.96 *SD

**Fig. 4:**
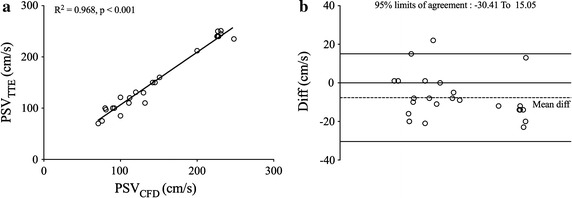
Validation of PSV derived from CFD. Demonstration of PSV and PSP validation. The image **a** was linear fitting of PSV, and image **b** was Bland–Altman plot of PSV. The reference line of Bland–Altman plots was mean difference ± 1.96 *SD

The data on PSP_CC_, PSV_TTE_, PSP_CFD_, and PSV_CFD_ are given in Table 3. PSP_CFD_ had an excellent correlation (Fig. 3a) with PSP_CC_ (r = 0.918, p < 0.001). The mean PSP_CC_ was 105.08 ± 15.38 mmHg, while mean PSP_CFD_ was 106.48 ± 15 mmHg. The mean bias was 1.405 mmHg (Fig. 3b, 95% confidence interval − 7.237–10.04). PSV_CFD_ was also excellently correlated with PSV_TTE_ (Fig. 4a, r = 0.968, p < 0.001). The mean PSV_TTE_ was 152.92 ± 64.36 cm/s, while mean PSV_CFD_ was 145.24 ± 61.68 cm/s. The mean bias was − 7.68 cm/s (Fig. 4b, 95% confidence interval − 30.41 to 15.05).

**Table 3:** Measured and simulated PSV and PSP

Case	PSV (cm/s)			PSP (mmHg)		
	TTE	CFD	R^2^	CC	CFD	R^2^
01	160	151	0.968	94	92	0.918
02	85	100		102	96	
03	100	80		123	120	
04	245	231		89	92	
05	130	130		133	137	
06	100	92		101	103	
07	120	112		121	126	
08	121	100		91	95	
09	240	226		92	98	
10	100	90		80	86	
11	100	80		88	93	
12	97	81		78	82	
13	110	111		118	114	
14	150	142		110	106	
15	131	120		110	114	
16	235	248		115	122	
17	150	145		81	84	
18	110	132		110	115	
19	75	76		115	110	
20	70	71		113	114	
21	212	200		117	121	
22	251	231		100	97	
23	250	227		110	102	
24	241	227		130	131	
25	240	228		106	112	

PSV_TTE_ and PSV_CFD_ were PSV measured by TTE and calculated by simulation respectively, while PSP_CC_ and PSP_CFD_ were PSP measured by CC and calculated by simulation

**Fig. 5:**
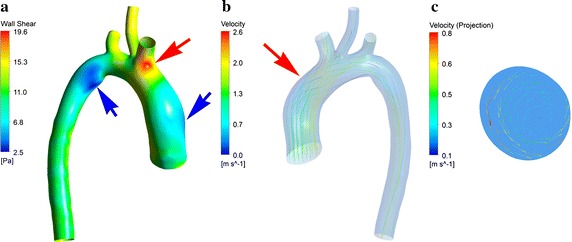
Distribution of PSWSS and streamline. Distribution of PSWSS was demonstrated in image **a**. Highest PSWSS was marked by red arrow, and lowest PSWSS was marked by blue one. Streamline at peak systolic was shown in image **b**, and the helical flow was marked by red arrow, and the projection of helical flow at cross section was demonstrated in image **c**

During peak systole, on the one hand, lowest PSWSS (2.83 ± 1.23 Pa) was observed at right wall of ascending aorta (AscAo) and inner wall of the attachment of arterial ductus ligament adjacent to AI. On the other hand, highest PSWSS (16.26 ± 3.43 Pa) was localized at outer wall of aortic arch, ostium of BA (Fig. 5). Streamline was also visualized and demonstrated in Fig. 5, velocity skewed toward the inner wall of AscAo and the region between inner and right wall of DAo (Fig. 5). Helical flow was observed at arch (Fig. 5), and there was highest velocity observed at ostium of BA (Fig. 5).

## Discussion

This study compared PSV_CFD_ with PSV_TTE_ and PSP_CFD_ with PSP_CC_ respectively. We demonstrated good agreements between reference standard method and CFD method.

Concerning the findings, there are additional factors that must be considered. Obviously, diameters of geometry reconstructed from MDCTA raw data will affect the CFD results, however, paired *t*-test found no difference between measured and reconstructed diameters, it means that geometry constructed from MDCTA raw data was identical to the real anatomy.

Given the results in the previous section, we observed that there are good agreements between PSV_TTE_ versus PSV_CFD_ and PSP_CC_ versus PSP_CFD_. The result demonstrated that CFD method was accurate comparing to TTE and cardiac catheterization. The biases of PSV and PSP were small, and there are many factors contributed to the biases. Data acquisition with MDCTA and catheterization was performed sequentially and thus there could be slight day-to-day variations in stroke volumes and other hemodynamics parameters [6]. More importantly, PSP was measured in sedated patients, whereas MDCTA and TTE were measured when patients awake. But according to the results, the biases were acceptable.

During MDCTA processing, there is a level of uncertainty when reconstructing the 3D aorta geometry based on 2D MDCTA raw data. Precise reconstruction is critical for the outcome of CFD simulation. However, no significant differences between measured and reconstructed geometric parameters were observed in this study.

The step of simulation may also be a factor contributing to the biases. Lumped parameter model was widely used to implement the pressure at outlet boundary according to previous studies [9, 14, 18, 25–27]. However, modeling hemodynamics in aorta with LPM involves a challenging set of constraints. In addition, previous studies have found that no-slip wall boundary is suitable and it is wildly used in vessel modeling. However, accurate wall boundary definition is still an active topic of investigation [28, 29]. In this work, second order no-slip wall boundary was included in the simulation, with the rational to reduce the computational effort and complexity of simulation.

In most of cases, low PSWSS of aorta was localized at outer or right luminal surface of ascending aorta or inner wall of the attachment of arterial ductus ligament adjacent to AI, while high PSWSS of aorta was located at outer wall of aortic arch, ostium of BA. This result corresponds to the distribution of WSS reported in other studies [14, 24]. In the current study, low PSWSS was observed at outer or right wall of AscAo maybe explained by the skewing velocity profile towards the inner wall during peak systole, while the velocity imposed to outer wall of AscAo was low. On the contrary, the highest PSWSS observed on outer wall of aortic arch and ostium of BA. This maybe explained by the arc shape of aortic arch. In this study, PSWSS on inner wall was lower than PSWSS on AI, and the larger diameter of arterial ductus ligament attachment as compared to AI maybe a factor contributed to this phenomenon.

Study conducted by Chiu et al. proved that PSWSS played an important role for regulating the arrangement and function of endotheliocyte [30]. Previous study also suggests that region with low PSWSS was correlated with areas of atherosclerotic plaque [31], and other study also indicated that excessive PSWSS was related with aneurysm formation of endothelial cells [32]. In addition, PSWSS also played an important role in the formation of aortic dissection (AD). Thubrikar reported that elevated PSWSS was correlated with sites of intimal tears [33], Wen also reported that initial location of tears was coincident with the region of maximal WSS [34], and Nordon reported that low PSWSS was benefit for minimizing the propagation of the dissection [35]. These studies indicated that PSWSS was related to the formation and prognosis of AD. Hence, WSS derived from the MDCTA raw data may provide not only anatomic information on aorta, but also additional information on plaque and dissection development.

Uniform distribution and smooth laminar blood flow was observed at AscAo, while skewing and helical flow was observed at aorta during peak systole. The flow pattern in current study was consistent with other studies [36, 37]. The inertial force was higher than viscous force during peak systolic, which help the development of helical flow, a usual physiological phenomenon in our cardiovascular system [38]. Helical flow in aorta exhibits important hemodynamic effect in increasing flux of oxygen [39] and achieving normal level of PSWSS [40]. Moreover, helical blood flow hinders the development of atherosclerotic plaques [23]. Similarly, the luminal surface low-density lipoprotein concentration in the aortic arch can also be reduced by helical flow [41]. Flow pattern generated by CFD based on MDCTA raw data may provide a reliable and convenient method to evaluate flow information.

### Study limitations

There were some limitations in the current study. First, the aortic valve morphology was not considered for its influence on the velocity profile. However, many studies have proven that the influence of valve morphology on velocity was small, and accurate simulation results had been achieved without considering aortic valvular morphology [14, 24, 42, 43]. Second, Constant LPM parameters were used to implement CFD in order to simplify the process of calculation despite unequal aortic morphology in each patient. However, their validated results suggested that the simulation error was negligible compared to other study [44, 45]. Third, this study had a limited number of subjects recruited in our study, and a follow up study with a larger number of subjects may provide a better valid assessment of aortic hemodynamic from MDCTA.

## Conclusions

Our results showed that CFD scheme based on MDCTA raw data is an accurate and convenient method in obtaining flow velocity and pressure from aorta and displaying the distribution of WSS and flow pattern of aorta. The preliminary results from our study demonstrated the capability in combining clinical imaging data and novel CFD tools in infants with CHD and provide t provide another noninvasive approach for diagnose of CHD such as CoA, AS or PAH in future.

## Abbreviations

CFD: computational fluid dynamics
TTE: transthoracic echocardiography
PSP: peak systolic pressure
PSV: peak systolic velocity
PSP_CFD_: PSP measured by CFD
PSV_CFD_: PSV measured by CFD
PSP_CC_: PSP measured by CC
PSV_TTE_: PSV measured by TTE
PSPG: peak systolic pressure gradient
BP: blood pressure
WSS: wall shear stress
PSWSS: peak systolic wall shear stress
LPM: lumped parameter model
CHD: congenital heart disease
MDCTA: multi-detector computed tomography angiography
CMR: cardiac magnetic resonance
PAH: pulmonary arterial hypertension
AS: aortic valve stenosis
CoA: coarctation of aorta
AscAo: ascending aorta
BA: brachiocephalic artery
LCCA: left common carotid artery
LSA: left subclavian artery
DAo: descending aorta
